# WO_3_/p-Type-GR Layered Materials for Promoted Photocatalytic Antibiotic Degradation and Device for Mechanism Insight

**DOI:** 10.1186/s11671-019-2975-1

**Published:** 2019-04-29

**Authors:** Wenfeng Zhao, Xiaowei Wang, Lizhe Ma, Xuanbo Wang, Weibin Wu, Zhou Yang

**Affiliations:** 1 College of Electronic Engineering, South China Agricultural University, Guangzhou, 510642 China; 2 College of Engineering, South China Agricultural University, Guangzhou, 510642 China; 3 School of Materials Science and Engineering, Nanyang Technological University, Singapore, 639798 Singapore

**Keywords:** Photo-induced doping effect, Layered materials, Photocatalytic dynamics processes, WO_3_/p-type-graphene, Antibiotic degradation, Mechanism insight

## Abstract

Graphene enhanced WO_3_ has recently become a promising material for various applications. The understanding of the transfer of charge carriers during the photocatalytic processes remains unclear because of their complexity. In this study, the characteristics of the deposited WO_3_/graphene layered materials were investigated by Raman spectroscopy, UV–vis spectroscopy, and SEM. According to the results, p-graphene exhibits and enhances the characteristics of the WO_3_/graphene film. The photocatalytic activities of WO_3_/graphene layered materials were assessed by the photocatalytic degradation of oxytetracycline antibiotics as irradiated by UV light. Here, a higher current of cyclic voltammetry and a higher resistance of impedance spectra were obtained with the as-grown WO_3_/graphene directly synthesized on Cu foils under UV light using an electrochemical method, which was different from traditional WO_3_ catalysts. Thus, it is urgent to explore the underlying mechanism in depth. In this study, a large layered material WO_3_/graphene was fabricated on a Si substrate using a modified CVD method, and a WO_3_/graphene device was developed by depositing a gold electrode material and compared with a WO_3_ device. Due to photo-induced doping effects, the current-voltage test suggested that the photo-resistance is larger than dark-resistance, and the photo-current is less than the dark current based on WO_3_/graphene layered materials, which are significantly different from the characteristics of the WO_3_ layered material. A new pathway was developed here to analyze the transfer properties of carriers in the photocatalytic process.

## Introduction

Collecting solar energy for generating electricity, one of the promising methods of smart and sustainable development, has aroused many research interests. For this end, photocatalytic water splitting generates hydrogen and oxygen from water, which plays an increasingly important role as the clean energy [1]. In this aspect, low-cost and high-efficiency photocatalysts are the typical representatives, e.g., WO_3_ and TiO_2_ [2]. Many reports showed that the formation of semiconductor composites can effectively obtain novel active photocatalyst systems because of the improvement of charge carrier separation [3]. Graphene (GR), the thinnest and strongest material, has many extraordinary chemical and physical properties for its unique two-dimensional structure with honeycomb carbon lattice. Graphene complex oxide semiconductor material, e.g., WO_3_/GR, was reported as one of the best photocatalysts in high-efficiency photoelectrochemical water splitting for its resilience to photocorrosion effect and efficient electron transport behaviors [4, 5]. Thus, graphene complex oxide semiconductor hybrid nanocomposite has aroused great research interest for its huge potential in the past decade for various applications, e.g., NO_2_ sensor, electrochromic materials, supercapacitor, and photocatalyst [6–12].

Given the superior photocatalytic performance of the WO_3_/GR, numerous studies have been conducted to reveal the underlying mechanism that graphene improves WO_3_ characteristics associated with photo-generated charge transfer, and several well-established explanations have been made. For instance, Wu et al. considered that the graphene can serve as an electron–acceptor material and reduce the recombination of photo-excited electron–hole pairs, thereby increasing photoconversion efficiency [13]. Furthermore, WO_3_ nanorods can provide another possible electron route between WO_3_ and coupled rGO nanosheets, thereby exhibiting excellent visible-light catalytic activity for hydrogen production and clarifying the Z-scheme catalytic mechanism [14–17].

Besides, a few experiments were performed to explain the mechanisms of oxide semiconductor materials and graphene hybrid nanocomposite [18, 19]. Pang et al. used the oxygen-18 isotope labeling technique as a powerful tool to analyze the complicated photocatalytic mechanisms on the TiO_2_ surface [20]. Recently, several groups reported that light can be used to achieve the charge doping in graphene, which can improve the understanding and the use of graphene Schottky junctions for optoelectronics and electronics [21, 22]. Moreover, photo-induced doping originates from a light-absorbing material on graphene heterostructure interfaces, and it has recently exhibited unique device characteristics and physical effects. Photogenerated charges from light–matter interaction are transferred to graphene, thereby leading to electronic structure tailoring in graphene. It is noteworthy that this non-contact doping approach, easy to control, will ensure no additional defects [23].

In this study, the layered materials WO_3_/GR were deposited, of which the characteristics were investigated under the Raman spectroscopy, UV–vis spectroscopy and SEM. All the results show that p-graphene emerges and improves the characteristics of the WO_3_/GR film. The photocatalytic activities of the layered materials were assessed by the photocatalytic degradation of oxytetracycline antibiotics under UV light irradiation. The characteristics of cyclic voltammetry and electrochemical impedance spectra of the as-grown WO_3_/GR directly fabricated on Cu foils under UV light using electrochemical behavior were obtained here and compared with traditional WO_3_ catalysts. To explore the charge transfer mechanisms associated with photo-induced doping, the stacks of large area layered materials WO_3_/GR were designed on the Si substrate using a modified CVD approach, and WO_3_/GR and WO_3_ devices were developed by depositing an electrode material of gold foil for comparison. The characteristics of WO_3_/GR were analyzed and compared with those of WO_3_ due to photo-induced doping effects using the current–voltage test. Charge transport behaviors of p-graphene can be modified to improve photocatalytic ability. Furthermore, graphene was used as the photogenerated electron acceptor and effectively suppressed the charge recombination in the WO_3_/GR layered materials.

## Experimental Section

Characterization of WO_3_/GR thin flake transistor: first, large-area graphene films of the order of centimeters were formed on copper substrates by chemical vapor deposition using methane. Graphene films were removed from the Cu foils to SiO_2_/Si substrate by etching in an aqueous solution of iron nitrate. The WO_3_ thin film was formed from 50 nm WO_3_ powder on a clean Si wafer with a 275-nm SiO_2_, graphene top layer [24]. During the deposition, argon was used as the protective gas. Subsequently, the electrodes (Cr/Au (5/50 nm)) were patterned with standard photolithography, electron beam metal deposition, and lift-off. For comparison, the pure WO_3_ device without graphene was prepared under the same conditions.

The band-gaps of the fabricated films were obtained by measuring absorbance using an UV–vis instrument (UV-2600, SHIMADZU Inc.). The morphology and microstructure of the nanostructured films were assessed with a JEOL JSM-7600F field emission scanning electron microscopy (FE-SEM). Raman measurements were performed in a Witec system in a backscattering configuration. The excitation was achieved by visible laser light (*λ* = 532 nm). All spectra were recorded at low power levels to avoid laser-induced modification or ablation of the samples.

Photocatalytic activity tests were performed under UV light. A defined amount of photocatalyst was suspended in 20 mL of antibiotic (oxytetracycline, 15 mg/L) solution in a typical activity test. The suspension was left in the dark for 1 h to reach the adsorption equilibrium, and the photocatalytic reaction was initiated under UV light for 160 min. The light source was a 250-W mercury lamp. By measuring the changes in the UV–vis absorption spectrum as a function of irradiation time, this study monitored antibiotic degradation.

### Electrochemical Measurements

All the electrochemical measurements were performed in a three-electrode system for CHI 604E electrochemical workstation (CH Instruments), in which WO_3_/GR/Cu foil and WO_3_/Cu foil served as the working electrode, Pt foil as a counter electrode, and a saturated Ag/AgCl as a reference electrode. All the potentials were calibrated by a reversible hydrogen electrode (RHE). Linear sweep voltammetry with a scan rate of ~ 0.1 V s^−1^, from + 0.20 to − 0.20 V vs. RHE was performed in 0.5 M H_2_SO_4_. The Nyquist plots were obtained at the frequencies ranged from 100 kHz to 0.1 Hz at the overpotential of 40 mV. To extract the series and charge-transfer resistance, the impedance data fitted to a simplified Randles circuit.

### Optoelectronic Measurement

All the electronic and optoelectronic characterization were performed in a probe station in a vacuum and at ambient temperature. The photocurrent was recorded by the Agilent 1500 A semiconductor analyzer. The light excitation was achieved by the 253 nm lamp used for the UV excitation.

## Results and Discuss

### The Characteristic of the WO_3_/GR Film

**Fig. 1:**
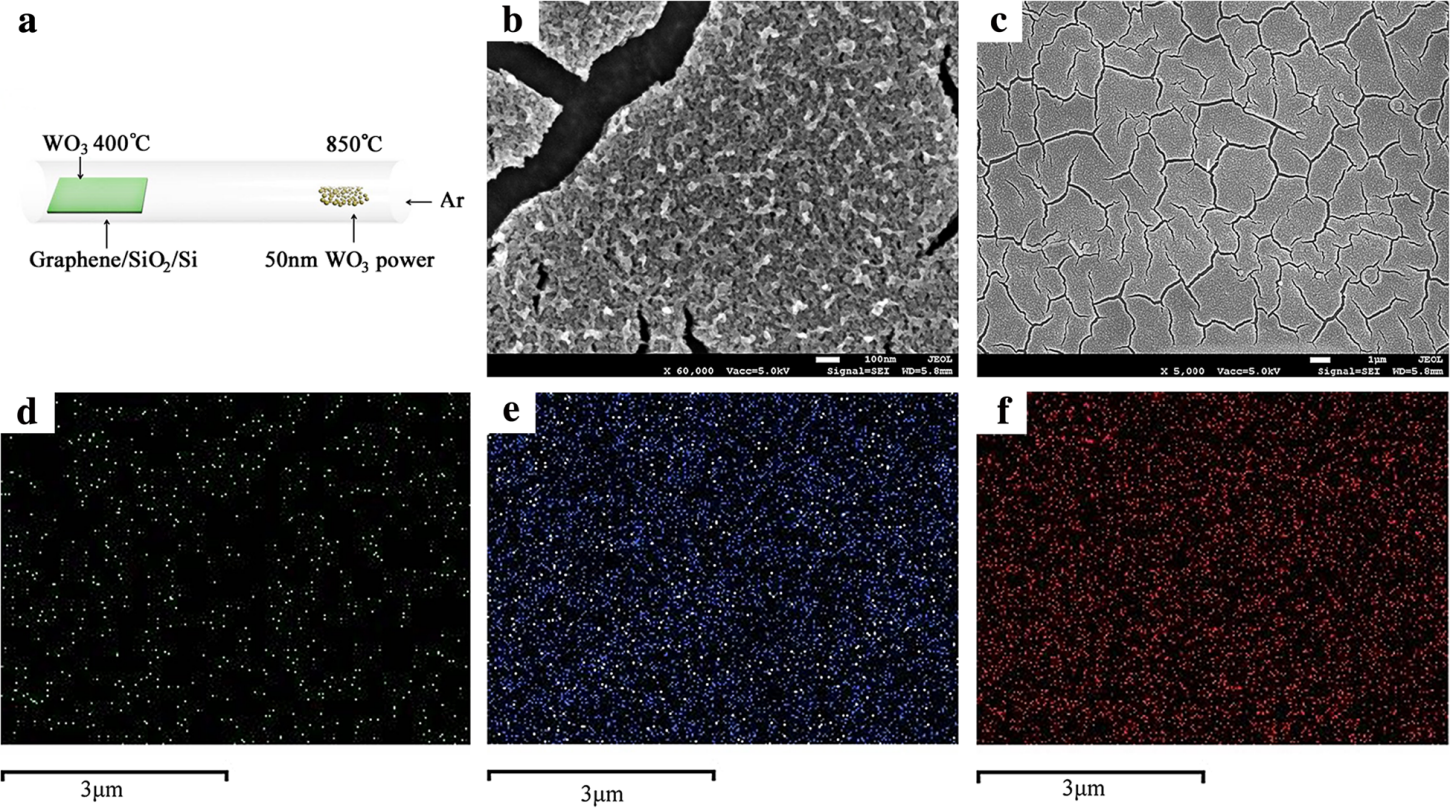
Schematic of the synthesis and the SEM morphologies of the WO_3_/GR heterostructures. **a** The 50 nm WO_3_ powder is positioned in the same ceramic boat at the inlet side of the tube furnace. **b** × 60,000 and **c** × 5000 SEM images. **d** C **e** O **f** WEDS elemental mapping of WO_3_/GR

The deposition process of WO_3_/GR and WO_3_ films by CVD is shown in Fig. 1a. Figure 1b and c give SEM photographs of the as-deposited WO_3_/GR thin films. It is found that the WO_3_/GR thin film materials are uniform and smooth here. Moreover, from inspection, small crack gaps about 100 nm in size were found on the surface of WO_3_/GR. Figure 1d, e, and f show the elemental mapping of C, O, and W on the WO_3_/GR surface. Obviously, both W and O are uniformly distributed over the surface with a higher percentage. Since graphene is grown below WO_3_, element C can be found at the position of crack gaps with a low percentage [25].

**Fig. 2:**
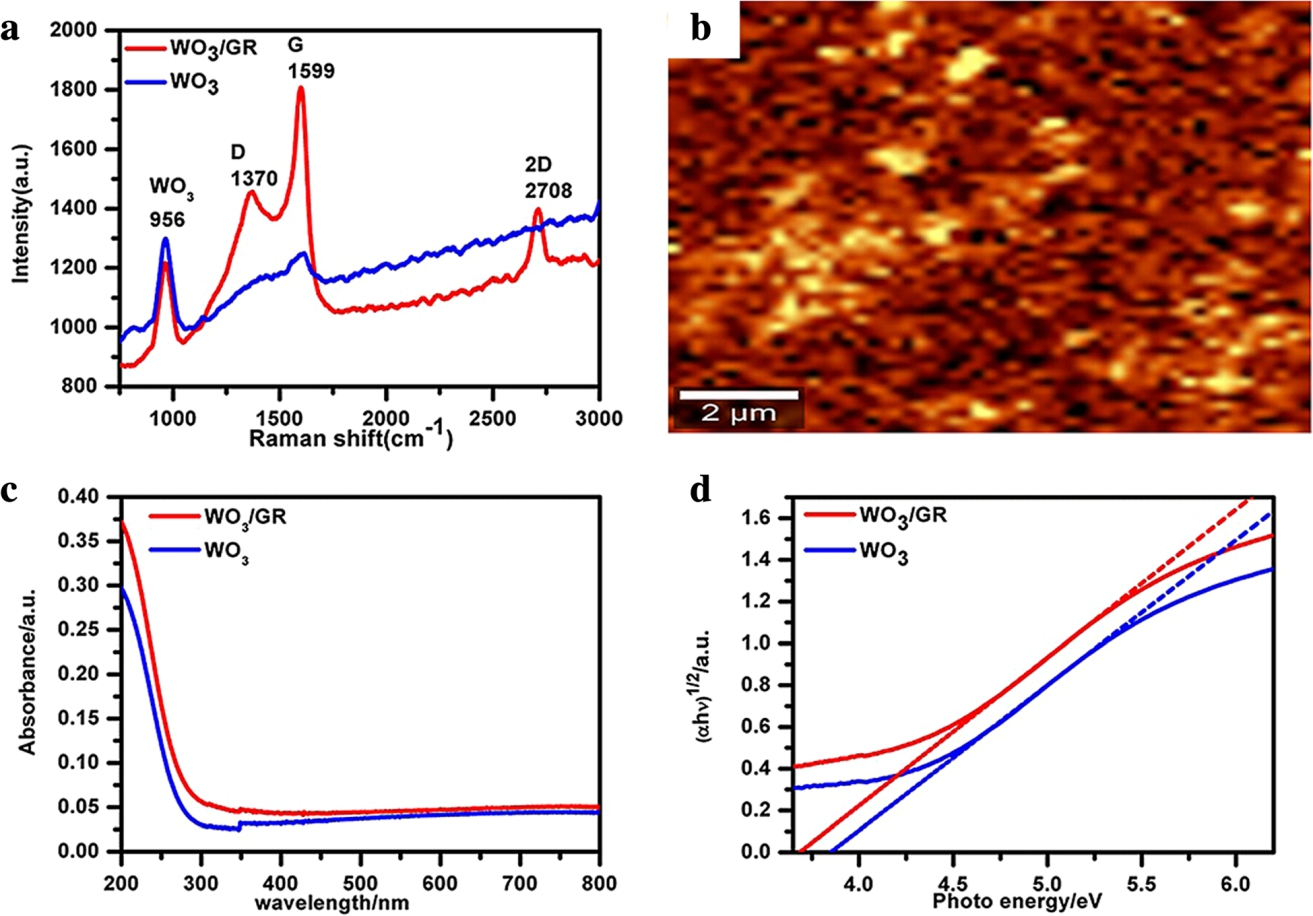
**a** The Raman spectra of as-prepared samples. **b** Raman G-peak mapping image of as-prepared samples. **c** UV–vis absorption spectra of as-prepared samples. **d** Determination the energy gap of samples

Figure 2a shows a selected region of the Raman spectra of the WO_3_/GR, as well as pure WO_3_. In general, monolayer graphene has two peaks at nearly 1348 cm^−1^ and 1586 cm^−1^, suggesting that the intensity ratio of I_G_/I_D_ peak is about 2 of a Raman spectrum. Similar peaks at D-band (round 1370 cm^−1^) and G-band (round 1599 cm^−1^) were observed in the WO_3_/GR composite. According to the spectra in Fig. 2a, the I_G_/I_D_ ratio decreased from 2 for the graphene to 1.2 for the WO_3_/GR composite. Thus, the smaller the I_G_/I_D_ peak intensity ratio of a Raman spectrum, the higher the defects and disorders of the graphitized structures will be in the WO_3_/GR composite due to the high temperature of nearly 400 °C. Due to the stretching mode O–W–O in the sample of WO_3_/GR composite, Raman vibrations centered at 815 cm^− 1^, the characteristic of pure WO_3_ was detected, which was constantly narrowed in the sample of WO_3_/GR composite. It is noteworthy that the G-band of WO_3_/GR had risen from 1584 to 1599 cm^−1^ compared with graphene. This G-band up-shift was the general evidence of chemical doping of carbon materials. The trend here is consistent with previous studies with the p-type doping of the graphene, leading to upshift of the G-band. According to the Raman G-band shift, charge transfer between the graphene and the WO_3_ in the WO_3_/GR composite was demonstrated [26, 27]. The 2D peak shifted to longer wavelengths, which also verifies that the graphene was effectively p-doped. The 2D band located at 2691 cm^−1^ for pristine (undoped) graphene and round at 2700 cm^−1^ for p-doped graphene, respectively [28].

The Raman data of WO_3_/GR composite were extracted into intensity mapping, and Fig. 2b shows the Raman G peak mapping image of the WO_3_/GR composites obtained from the G-band of the graphene. The “bright” regions with high intensity illustrate the presence of the graphene, and it can be confirmed that p-doped graphene and defects exist in the layered materials due to the local high bright regions. Also, the “dark” regions are related to the WO_3_ information, which present the large area distribution of the graphene in the layered materials [29].

UV–vis spectra were treated as a key method to obtain the light absorption properties of photocatalysts. To analyze the interaction of graphene and WO_3_, UV–vis absorption spectra were recorded as shown in Fig. 2c. The equation αhʋ = *A* × (hν-Eg)^*n*/2^ was used, where α, ν, Eg, and *A* are the absorption coefficient, the light frequency, the band gap, and a constant, respectively [30]. The (αhν)^1/2^-hν curves of as-prepared samples are shown in Fig. 2d. According to the results, the light absorption of WO_3_/GR in the visible light region was more sensitive than that of pure WO_3_. The mixture of graphene onto the WO_3_ improved the absorption capacity to the light. Compared with pure WO_3_, the band gap of WO_3_/GR was narrowed from 3.88 to 3.68 eV (Fig. 2d). According to the redshift and enhancement of light absorption, WO_3_/GR exhibits the improved activity to separate electrons and holes.

### The Degradation of Antibiotics Oxytetracycline

**Fig. 3:**
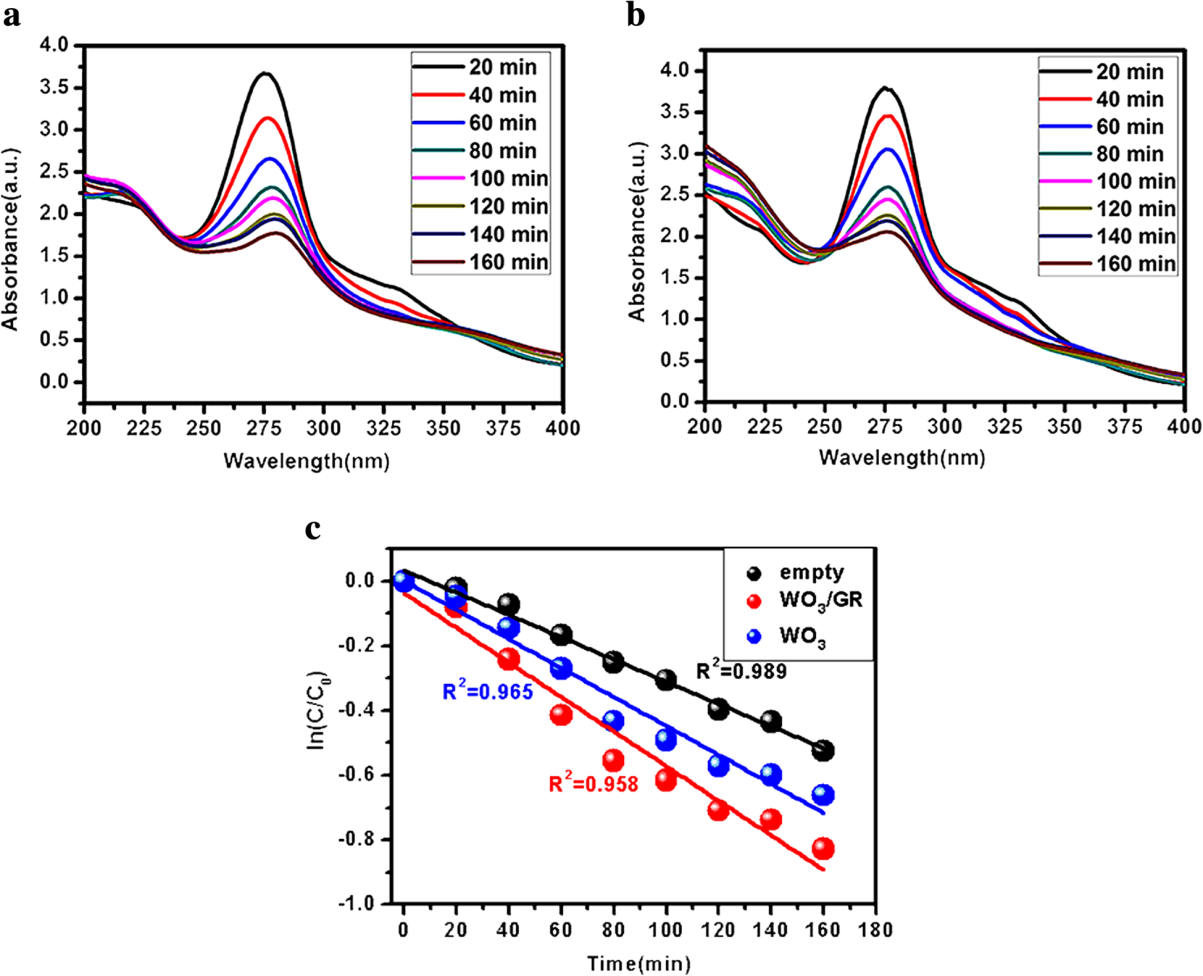
**a** UV–vis spectra of the degradation of antibiotics in the presence of WO_3_ composites. **b** UV–vis spectra of the degradation of antibiotics in the presence of WO_3_/GR composites. **c** Kinetics of as prepared WO_3_ and WO_3_/GR

The detailed roles connected with doped graphene in oxide semiconductor photocatalysts appear to be complicated so that more work in fundamental researches is developed following this direction. The photocatalytic abilities of graphene-based photocatalysts can be improved by strengthening both electronic conductivity and carrier mobility. The conductive graphene can receive the photo-excited electrons as reservoirs when coupling graphene and the semiconductors. Accordingly, the concentration of photo-excited electrons decreased in semiconductor, thereby significantly suppressing their reductive corrosions [31]. Photocatalytic activity and reaction kinetics of WO_3_/GR, WO_3_ were observed during the degradation of antibiotics oxytetracycline using UV light (365 nm) as shown in Fig. 3. The photocatalytic activity of composite with photocatalyst and without photocatalyst was determined here in UV light for the comparison. After a specific time interval under UV light, the peak intensity of oxytetracycline associated with the UV–vis absorption characteristics of oxytetracycline molecule at 275 nm gradually decreased after 160 min as shown in Fig. 3a and b. Compared with WO_3_, WO_3_/GR led to a high degradation of oxytetracycline. The kinetics of oxytetracycline degradation under UV light can be obtained by pseudo-first-order reaction, where C0 and C are initial and concentration at given degradation time *t* and *k* is the rate constant, respectively. The diagram of ln(C/C0) was plotted as a function of *t* (Fig. 3c).

$$ \mathrm{In}\left(\mathrm{C}/{\mathrm{C}}_0\right)= kt $$

The graph for WO_3_/GR, WO_3_ fitted linearly, where the correlation coefficient of *R*^2^ and the value of rate constant *k* (*k*_empty_ =  − 0.0034 min^−1^, ${k}_{{\mathrm{WO}}_3}=-0.0045\ {\min}^{-1}$, ${k}_{{\mathrm{WO}}_3/\mathrm{GR}}=-0.0054\ {\min}^{-1}$) show the higher catalytic activity of WO_3_/GR in comparison with WO_3_. It is because the formation of heterojunctions promotes the separation of electrons and holes. Holes can generate ^•^OH, which is considered the major reactive species for the oxidation reactions.

### Electrochemical Behavior of the Layered Materials

**Fig. 4:**
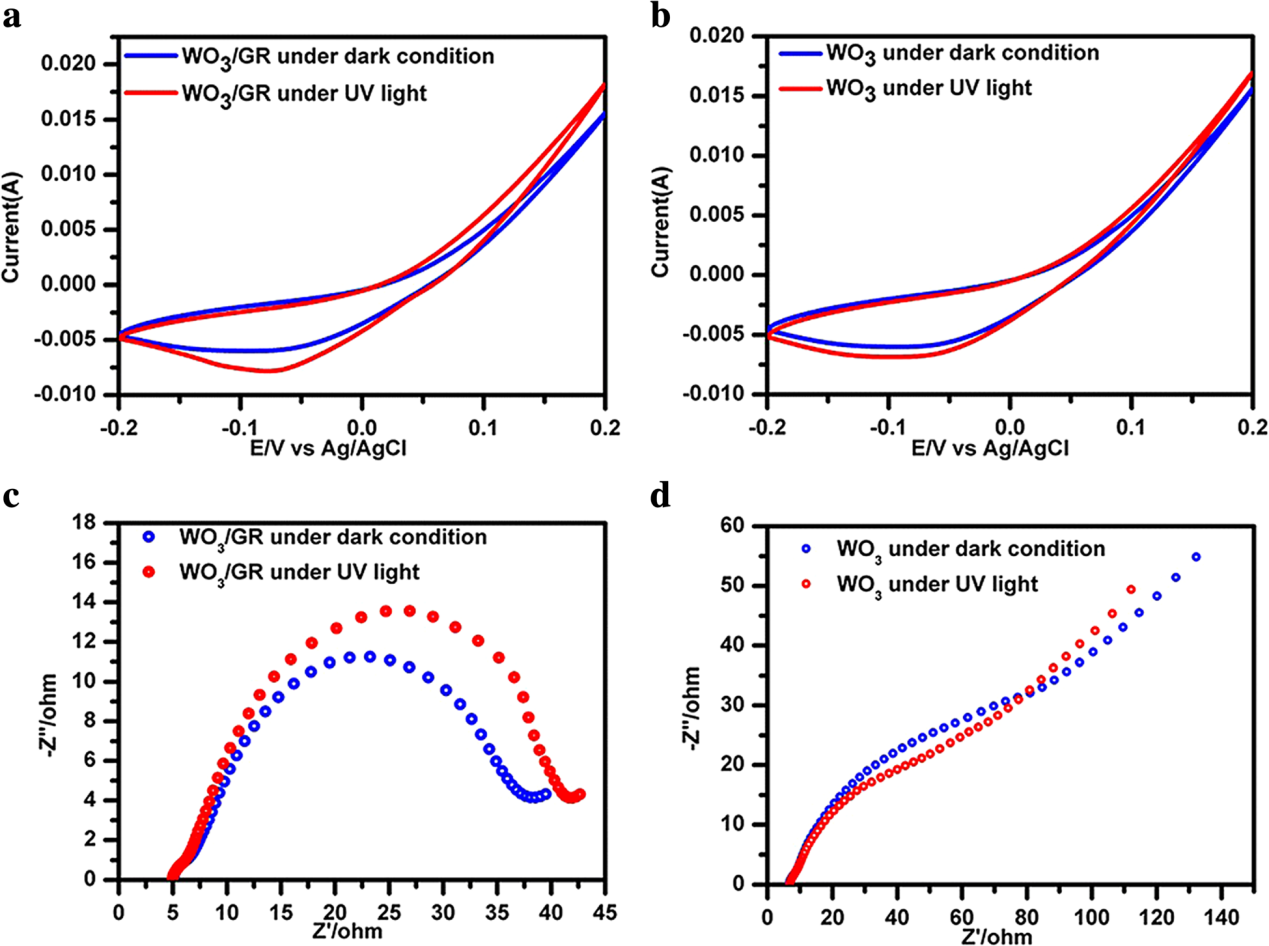
Electrocatalytic application of CVD synthesized layered materials WO_3_/GR and WO_3_. **a**, **b** CV curves of as-grown WO_3_/GR, WO_3_ on Cu foil. **c**, **d** electrochemical impedance spectra of WO_3_/GR, WO_3_ flakes as well as the Cu foil substrate

Cyclic voltammetry is considered the method of analysis of the photoelectrocatalytic characteristics of WO_3_/GR/Cu and WO_3_/Cu electrodes for the reduction of hydrogen, as shown in Fig. 4a and b. Under the action of UV light, the current of the Cu electrode under ultraviolet light (8.5 mA) is larger than that in the dark (4 mA). The current of WO_3_/Cu electrode showed a slight difference between a dark condition and UV light. Moreover, WO_3_/GR/Cu electrode showed lower overpotential at − 0.08 V than WO_3_/Cu electrode at − 0.06 V. Reduction of hydrogen from the catalyst generated the response WO_3_ redox site. According to all the above results, it was clear that WO_3_/GR/Cu electrode was more efficient and showed the enhanced functional properties compared with that of WO_3_/Cu. This suggested that the presence of graphene under UV light led to the lower potential value and increased reduction currents under photo-induced doping effects which excited more electrons from WO_3_ to graphene.

The interfacial characteristics of the modified electrode, which were of huge significance to the electrical conductivity, and the electrocatalytic properties of the modified electrode were analyzed here by EIS. The electron-transfer kinetics and diffusion characteristics can be concluded from the shape of the electrochemical impedance spectrum. The semicircular portion, Ret, obtained at higher frequencies represents an electron transfer-limited process, and the linear portion at lower frequencies was attributed to the limited mass transfer of the as-prepared sample ion [32, 33]. Figure 4c and d show the results of EIS for electrodes of WO_3_/GR/Cu and WO_3_/Cu. WO_3_/GR/Cu electrode shows a better depressed semicircle arc compared with the WO_3_/Cu electrode, representing an excellent diffusion electron-transfer process on the WO_3_/GR/Cu electrode surface. Under UV light, WO_3_/Cu electrode still shows the lower depressed semicircle arc (Ret of 50(Z′/Ω)) compared with Ret (75(Z′/Ω)) in the dark. Note that under the UV light, WO_3_/GR/Cu electrode shows a relatively obvious semicircle arc (Ret = 42(Z′/Ω)), indicating a higher electron transfer resistance behavior than that of Ret (38(Z′/Ω)) in the dark. The increase in the value of electron transfer resistance (Ret) due to photo-induced doping effects improved Fermi energy level of graphene on electrode surface under UV light. These results also demonstrated that the graphene can improve the electron transfer rate between the electrode and WO_3_, which is consistent with the CV results.

### The Charge Transfer Behaviors from WO_3_/GR Composite Device

**Fig. 5:**
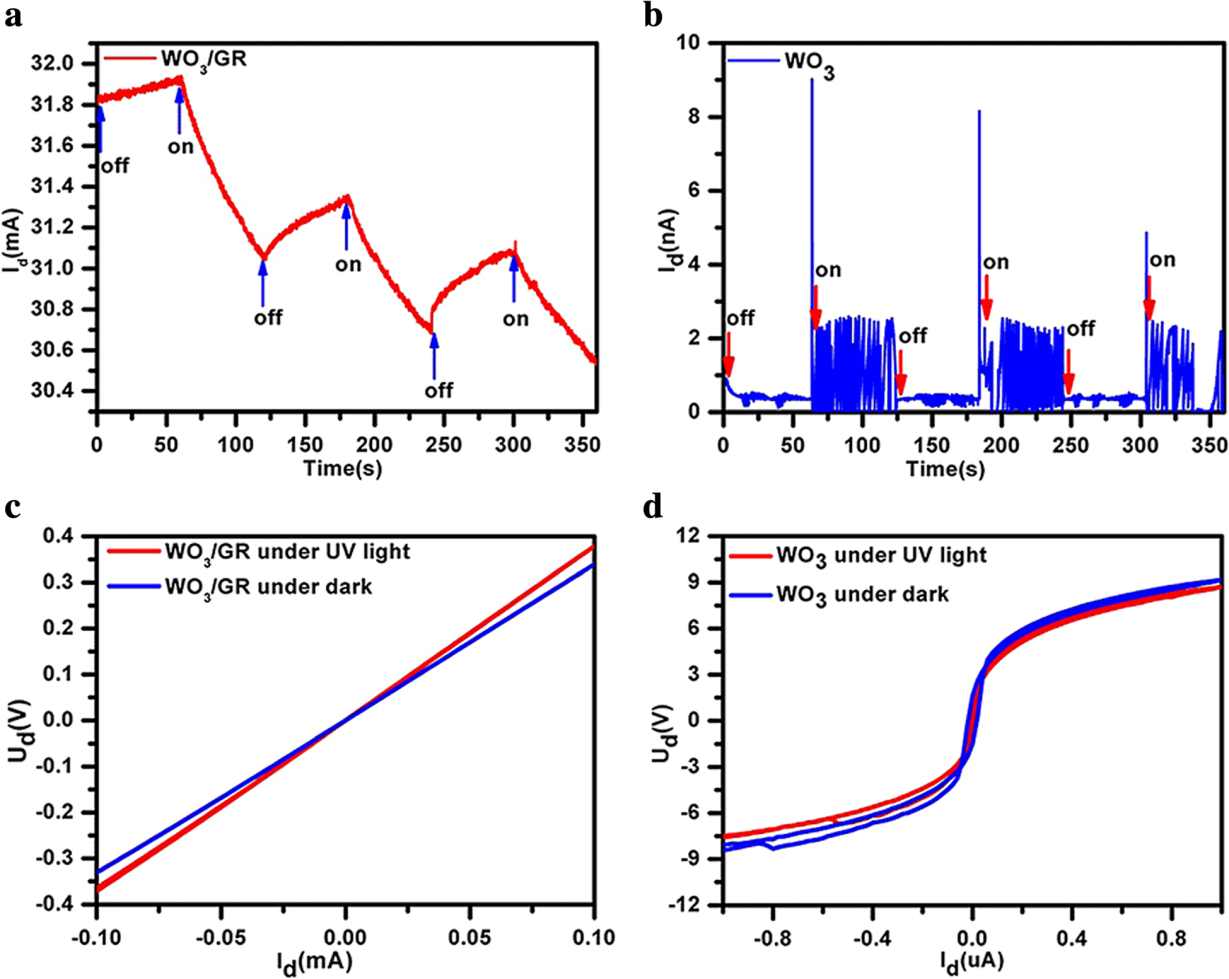
Experimental observation of characteristics in WO_3_/GR device compared with the pure WO_3_ device. **a** Photocurrent of WO_3_/GR. **b** Photocurrent of WO_3_. **c** Photoresistance of WO_3_/GR. **d** Photoresistance of WO_3_

Charge transfer behaviors in the WO_3_/GR layered materials can be surveyed under UV light, as shown in Fig. 5. The typical I–V and I–T characteristics of the device fabricated from WO_3_/GR composite and the reference device with pure WO_3_ were measured in the dark and under UV light at 253 nm with the intensity of 0.3 mW/cm^2^ as shown in Fig. 5a and b [34]. The photocurrent of the WO_3_/GR composite device was nearly 106 times higher than that of the reference device from pure WO_3_. Note that the photocurrent was less than the dark current of the WO_3_/GR composite, which is significantly different from the reference device from pure WO_3_. The typical I–V characteristics of the device were similar to I–T characteristics (Fig. 5c, d). The WO_3_/GR resistance R with optical illumination was larger than that in the dark due to photo-induced doping effect. The WO_3_/GR resistance R showed a constant value about thousands of ohms with optical excitation and dark conditions. However, the reference device, pure WO_3_ resistance still showed essential semiconductor features [35].

**Fig. 6:**
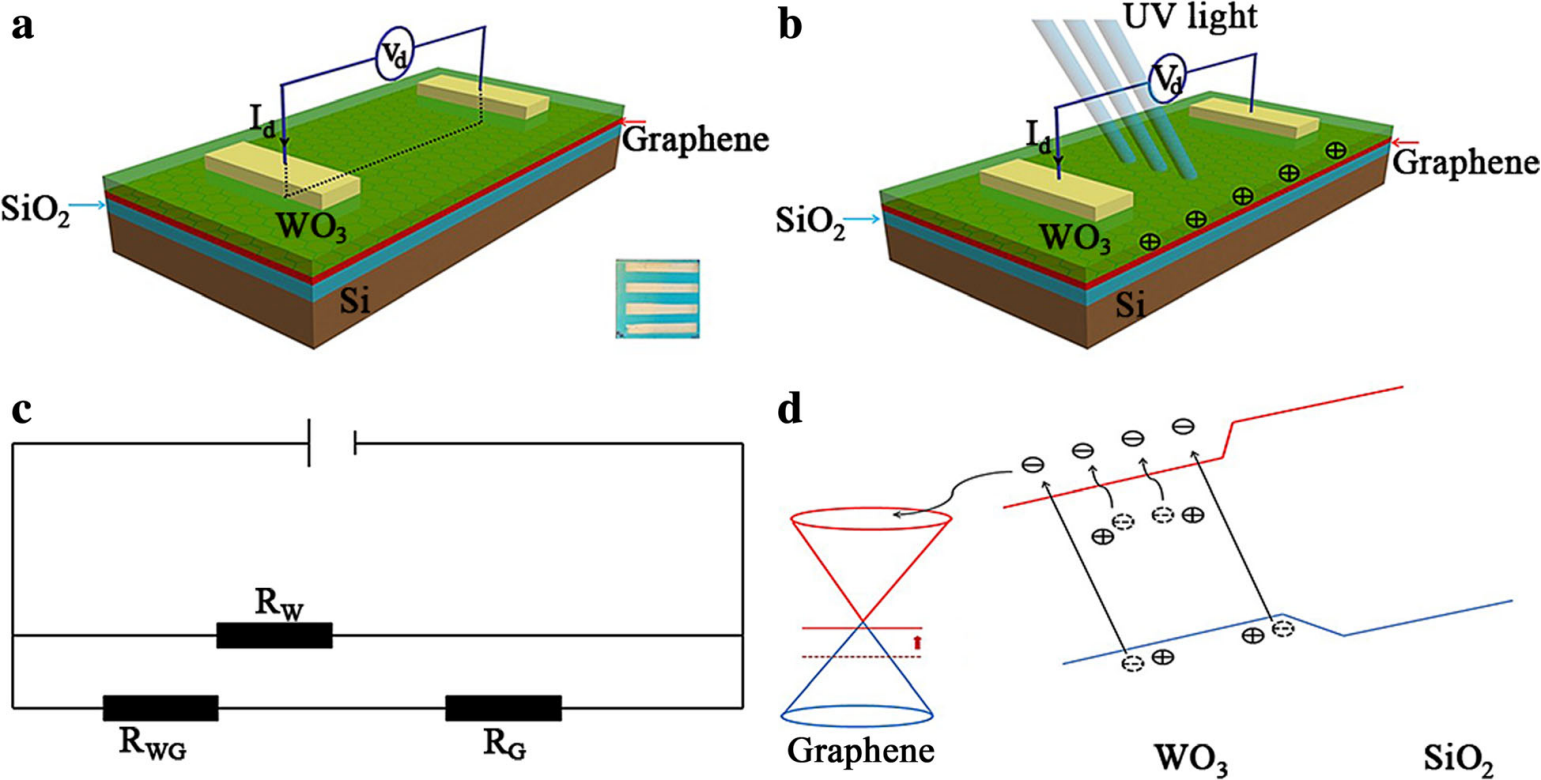
Characteristics of WO_3_/GR after photo-induced modulation doping. **a**, **b** Current route and charge distribution in the WO_3_/GR device in case of UV light. Positive charges accumulate in WO_3_ under light illumination. Yellow, Cr/ Au; green, WO_3_; red, graphene; blue, SiO_2_; gray, Si. **c** Equivalent circuit model of the WO_3_/GR device. **d** Schematics of the band structure of the WO_3_/GR heterostructure and illustration of the photodoping mechanism, in which optical excitation first excites electrons from defects in WO_3_. Red (blue) lines represent the conduction (valence) band. The excited electrons enter the graphene, and the positively charged defects lead to modulation doping in the graphene

Figure 6 shows the characteristics of WO_3_/GR after photo-induced modulation doping. Current route and charge distribution in the WO_3_/GR device under UV light are shown in Fig. 6a and b. Positive charges accumulated in WO_3_ under illumination. The higher current of the WO_3_/GR composite device should be attributed to the improved conductivity of the composite through GR. Graphene can create a Schottky contact at the interface with WO_3_, thereby forming resistance *R*_WG_ [36]. The device can be modeled by the circuit as shown in Fig. 6c. Due to WO_3_ resistance *R*_W_>>(*R*_WG_ + *R*_G_), the current of the device was decided by *R*_WG_ + *R*_G_. Therefore, the conductivity properties had been significantly improved in the presence of graphene.

Schematics of the band structure of the WO_3_/GR hybrid composites and diagram of the photo-induced doping mechanism are shown in Fig. 4d. The WO_3_/GR heterostructure device without light illumination is consistent with the previous result of a stable p-type doped graphene transistor, in which electrons were transferred from the graphene thin film to WO_3_. Initially, graphene was hole-doped in the dark, and an electric field appeared from graphene to silicon. As shown in Fig. 6d, when the device was under UV light, on the one hand, the electrons in the valence band (VB) of WO_3_ were excited to the conduction band to create electron–hole pairs [37–39]. On the other hand, electrons of donor-liked defects in WO_3_ were excited by photons to the conduction band. The ionized defects were positively charged and localized in the WO_3_. These excited electrons in both cases can be mobile, move towards, and then enter the graphene. It was suggested that significant photo-induced electron transfer occurred from WO_3_ to graphene at the WO_3_/GR device [40].

The excited electrons entered the graphene, and the positively charged defects led to modulation doping in the graphene. Under this modulation doping in the graphene, WO_3_/GR heterojunction emerged. Subsequently, the experimental data shows a decrease in conductivity with the increase in Fermi energy, EF of graphene, thereby leading to a slow decrease in the UV photocurrent. This is well consistent with the theoretical model [41]. It is therefore suggested that the transport behavior of the device will be utterly different from pure WO_3_ when the WO_3_/GR device is exposed to light. Photo-induced doping effects were also reported by some authors. Tiberj et al. reported that the charge carrier density of graphene can be finely and reversibly tuned between the hole and electron doping due to photo-induced doping, which was significantly affected by the substrate cleaning method [42]. Ju et al. showed photo-induced doping can maintain the high carrier mobility of the graphene/boron nitride heterostructure [43].

Under the light-induced doping effect, the surface of WO_3_/GR, as the primary photosensitive particles, has more photogenerated holes than the pure WO_3_ surface under UV light. The more active sites of the WO_3_/GR surface pores, the more efficient the improvement of photosensitivity [44]. In general, the conductive graphene, as an electron transport mediator, could extend the lifetime of photogenerated charge carriers significantly and strengthen charge extraction and separation. For instance, Weng et al. assembled the graphene−WO_3_ nanorod nanocomposites, which improved the visible-light photocatalytic performance compared with bare WO_3_ nanorods [45, 46]. Therefore, how to enhance the photodegradation process of photo-induced doping by doping graphene should be explored. It may be related to the intensity of UV light, dopant concentration, and so on [47, 48]. Chu et al. fabricated GR–WO_3_ composites mixed with different amounts of graphene (0, 0.1, 0.5, 1, and 3 wt%). Moreover, the sensor based on 0.1 wt% GR–WO_3_ composite exhibits good selectivity and high response in comparison with those of pure WO_3_ [49, 50]. It may be based on the reason that the excessive proportion of graphene absorbed on the surface of WO_3_, decreasing the amount of the active sites. Subsequently, the proper proportion of WO_3_ and graphene can gain the best experimental effect. Akhavan et al. also analyzed the characteristics of TiO_2_/GO (graphene oxides) sheets at different irradiation times [51]. They found the GO can be photocatalytically reduced, and the carbon defects increased under irradiation, which was considered to be partly because of photo-induced doping here [52]. Accordingly, this study develops a new route for exploring carrier transfer behaviors and photo-induced doping effects in graphene-based photodegradation materials.

## Conclusion

In this study, the photocatalytic activities of the layered materials were assessed by the photocatalytic degradation of oxytetracycline antibiotics under UV light. A higher current of cyclic voltammetry and large resistance of impedance spectra with the as-grown WO_3_/GR directly synthesized on Cu foils under UV light through electrochemical behavior were obtained, which was also different from traditional WO_3_ catalysts. The characteristics of WO_3_/graphene layered materials were investigated under the Raman spectroscopy, UV–vis spectroscopy, and SEM. All results show that p-graphene emerges and enhances the characteristics of the WO_3_/GR film. The stacks of large-area WO_3_/GR layered materials were designed on the Si substrate using a modified CVD approach, and WO_3_/GR and WO_3_ films were fabricated on an electrode material of gold foil for comparison. Due to photo-induced doping effects, the current–voltage test suggested that the photo-resistance was larger than dark-resistance, and photocurrent was less than dark current based on WO_3_/GR layered materials, which were different from the characteristics of WO_3_ layered materials. Besides, charge transport behaviors of p-graphene could be modified to improve photocatalytic ability. Graphene serves as the photogenerated electrons acceptor and effectively suppresses the charge recombination in the WO_3_/GR layered materials. This study is considered a significant advance towards unraveling photocatalytic dynamics processes based on graphene and oxide semiconductor. Hopefully, these results can motivate scientists to explore high efficient catalysts for related applications.

## Abbreviations

CVD: Chemical vapor deposition
EF: Fermi energy
GO: Graphene oxides
GR: Graphene
IG/ID: D peak to G peak intensity ratio
RG: Resistance of graphene
rGO: Reduced graphene oxides
RW: Resistance of WO_3_
RWG: Resistance of WO_3_/graphene
SEM: Scanning electron microscope
UV: Ultraviolet
VB: Valence band
